# A Review of the Preparation, Analysis and Biological Functions of Chitooligosaccharide

**DOI:** 10.3390/ijms19082197

**Published:** 2018-07-27

**Authors:** Shuang Liang, Yaxuan Sun, Xueling Dai

**Affiliations:** 1 Beijing Key Laboratory of Bioactive Substances and Functional Foods, Beijing Union University, Beijing 100191, China; 171083210405@buu.edu.cn; 2 Department of Food Sciences, College of Biochemical Engineering, Beijing Union University, Beijing 100023, China; sunxx@buu.edu.cn

**Keywords:** chitin, chitosan, chitooligosaccharide (COS), COS preparation, COS analysis, COS biological activity

## Abstract

Chitooligosaccharide (COS), which is acknowledged for possessing multiple functions, is a kind of low-molecular-weight polymer prepared by degrading chitosan via enzymatic, chemical methods, etc. COS has comprehensive applications in various fields including food, agriculture, pharmacy, clinical therapy, and environmental industries. Besides having excellent properties such as biodegradability, biocompatibility, adsorptive abilities and non-toxicity like chitin and chitosan, COS has better solubility. In addition, COS has strong biological functions including anti-inflammatory, antitumor, immunomodulatory, neuroprotective effects, etc. The present paper has summarized the preparation methods, analytical techniques and biological functions to provide an overall understanding of the application of COS.

## 1. Introduction

**Figure 1:**
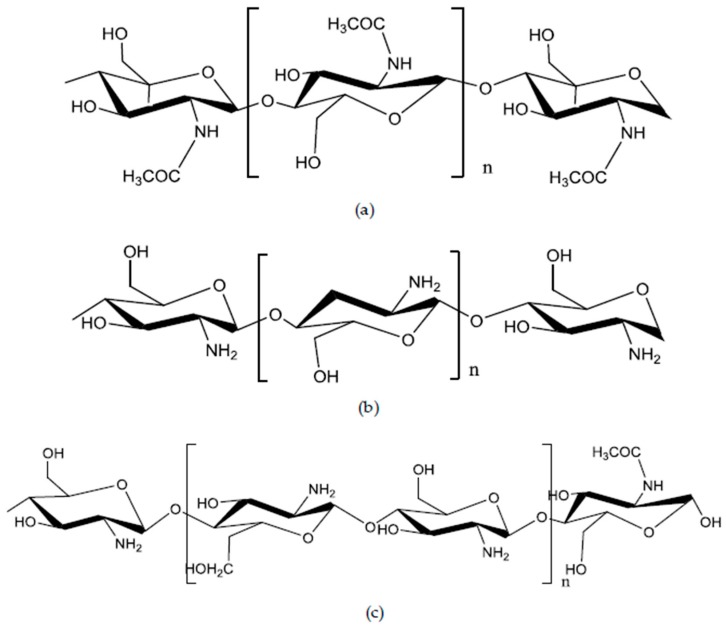
Structure of chitin (**a**); chitosan (**b**) and chitooligosaccharide (*n* ≤ 9, **c**).

Chitin, also named poly (β-(1-4)-*N*-acetyl-d-glucosamine), is the most abundant polymer other than cellulose and the main sources are exploited from two marine crustaceans, shrimp and crabs [1,2]. For industrial production, chitin is extracted from crustaceans by two steps: deproteination with aqueous NaOH and demineralization with HCl [3]. Chitin has three allomorphs, including α-, β-, and γ-chitin, which are distinctive in size in terms of the unit cell and degree of hydration. α- and β-chitin are constituted by layers of polysaccharide chains with parallel and anti-parallel structures, respectively. However, γ-chitin contains parallel polysaccharide chains, scattered with antiparallel single chains [4]. Chitosan is an essential deacetylated product of chitin, consisting of more than 80% β-(1,4)-2-amino-d-glucopyranose and less than 20% β-(1,4)-2-acetamido-d-glucopyranose [5]. Both chitin (Figure 1a) and chitosan (Figure 1b) are extensively used in cosmetics, medicine and water treatment due to their non-toxicity, biocompatibility and bioactivity [6,7,8,9,10]. Recently, Siddaiah et al. [11] designed chitosan nanoparticles (CNP) with low-molecular-weight chitosan as well as a higher degree of acetylation and explored the effect of CNP on pearl millet downy mildew; the results indicated that CNP were able to induce systemic and durable resistance against *Sclerospora graminicola* by improving the generation of nitric oxide. However, poor solubility at neutral pH has limited the application of high-molecular-weight chitin and chitosan.

**Scheme 1:**
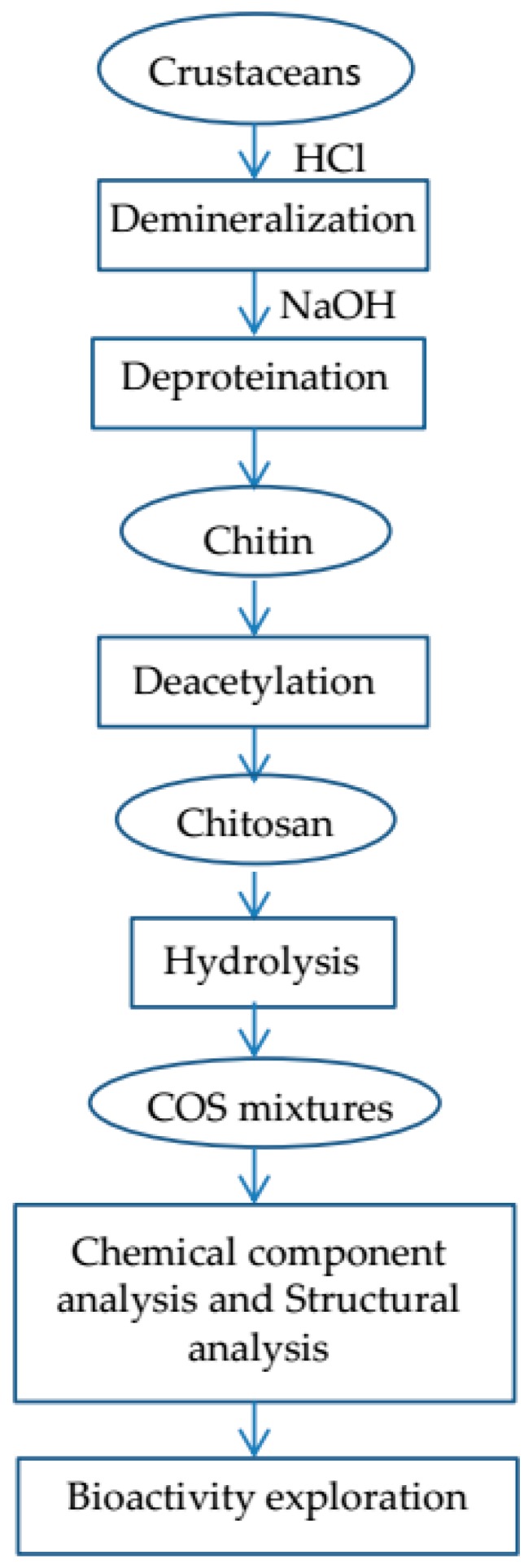
Chitooligosaccharide preparation and analysis process.

Chitooligosaccharide (COS, Figure 1c), also called chitosan oligomer or chitooligomer, is defined as chitosan with a degree of polymerization ≤20 and an average molecular weight of less than 3900 Da (generally 0.2–3.0 kDa) [12,13]. Sometimes much larger molecules are also called COS, and there can be very large differences in the activity of short (0.2–0.8 kDa) versus long (2.0–3.0 kDa) oligomers. COS has the advantages of low molecular weight, high solubility, good moisture absorption, strong absorption capacity, and good biocompatibility, etc. Besides, COS has potential ability in promoting food quality and human health and can also prevent the growth of bacteria and fungi [14,15], exert anti-tumor activity [16,17], alleviate inflammatory response [18,19], and act as an immunopotentiator [20,21,22]. COS should be stored under inert conditions and below −20 °C for long-term storage due to its sensitivity to autooxidation. The shelf life of COS is prominently prolonged when antioxidants like vitamin C or salts like sodium chloride are stored with COS [23]. In this review, we will provide an up-to-date overview of the preparation, analysis and biological activity of COS. COS preparation and analysis process are presented in Scheme 1.

## 2. Preparation of Chitooligosaccharide

At present, the preparation methods of COS mainly include chemical methods (hydrogen peroxide oxidation, acid hydrolysis), physical methods (microwave treatment, ultraviolet radiation, and ultrasonic treatment), enzymolysis, electrochemical method and composite degradation methods derived from these methods. Both advantages and disadvantages of various preparation methods are summarized in Table 1.

### 2.1. Chemical Degradation

The chemical degradation method is simple to handle, but the relative molecular weight of the degradation products is widely distributed, and therefore it is difficult to separate and purify the products. Besides, the consumption of reagents is considerable, and the post-treatment is complicated. The main methods involved in chemical degradation of COS include acid degradation and oxidative degradation. Jia et al. prepared low-molecular-weight chitosan using 85% phosphoric acid catalyzing as well as appropriate heating acceleration, and the result proved that low-molecular-weight chitosan, which is meaningful for improving solubility, could be obtained by increasing hydrolysis time [24]. Trombotto et al. reported another acid hydrolysis method to prepare COS. First, fully *N*-deacetylated chitosan was depolymerized by concentrated HCl (~12 M); as a result, a low degree of polymerization glucosamine (GlcN) oligomers could be produced. Second, activated charcoal was used to bleach products followed by the removal of excess HCl by co-vaporization. Third, the lowest degree of polymerization oligomers were decreased by hyperfiltrating the hydrolysate over a 500 g/mol cutoff cellulose acetate membrane followed by absolute ethanol precipitation and the highest degree of polymerization oligomers werecleared through subsiding by adjusting the pH of the hydrolysate to 8–9 via the addition of concentrated NaOH. Finally, the expected mixtures of a low degree of polymerization GlcN oligomers that account for around 7.5% of the weight of raw material could be produced with the help of centrifugation or freeze-drying [25]. Nitrous acid could also be used to prepare COS [26,27]. Moreover, the oxidative degradation method could be used in COS preparation where hydrogen peroxide, ozone, sodium perborate and potassium persulfate were employed as oxidants [28]. Xia et al. used hydrogen peroxide and phosphotungstic acid to prepare water soluble chitosan with the most applicable conditions as follows: 2% H_2_O_2_ (*v*/*v*), 0.1% phosphotungstic acid (*w*/*v*), 40 min, and 65 °C. The final products were white powders with a degree of polymerization of approximately 7 [29].

### 2.2. Physical Degradation

COS prepared by physical degradation is much easier to maintain the purity. Ultrasonic, microwave, and gamma rays could be used to prepare low-molecular-weight chitosan with little contamination but low productivity. Baxter et al. utilized acetic acid to treat chitosan with ultrasonic assistance. It is suggested that ultrasonic energy in the low to medium power range could take place of chemical and enzymatic methods which are used to modify the molecular weight of chitosan [30]. Ronge et al. reported that hydrolysis could be accelerated by addition of inorganic salts in a microwave irradiation field [31]. Besides, Co-60 γ-rays could lower the molecular weight of chitosan and the purity is satisfactory though there is a browning effect of chitosan, and it almost does not change the chemical qualities of chitosan such as the functional groups [32]. Hydrodynamic cavitation is a prospective method to degrade organic polymers that can be operated by passing the liquid through a constriction such as orifice plate, which leads to higher bubble densities, better scale-up probability, and lower expenditures [33,34]. Swirling cavitation is one kind of hydrodynamic cavitation, and Wu et al. degraded chitosan by swirling cavitation driven by a turbine. It has been proved that degradation efficiency increased with increased time, pressure, temperature and decreased solution concentration. The optimum conditions were as follows: solution concentration of 1 g·L^−1^, at 0.4 MPa, 60 °C, for 3 h. Under these conditions, the original viscosity reduction rate of chitosan was 83.65% [35].

### 2.3. Enzymatic Degradation

The enzymatic degradation method is apparently easier to operate and monitor than acid hydrolysis, and the products can be gained without additional modifications. However, the cost, availability and specificity of chitosanase have limited its application [36]. Enzymatic hydrolysis can be classified into specific enzymatic hydrolysis (chitinase, chitosanase, glucanase, etc.) and non-specific enzymatic hydrolysis (lysozyme, protease, lipase, amylase, cellulose, etc.) [37]. *Bacillus alvei* could produce an enzyme named extracellular enzyme preparation (EEP), and when chitosan was treated with EEP, the molecular weight of chitosan was decreased from 88,000 to 5000 Da, and the viscosity almost approached zero within 2 h. The optimum conditions of EEP were as follows: pH 5.5, 37 °C, 2 h and the concentration of chitosan solution was 1% [38].

COS can also be prepared by commercial α-amylase. Raw material prepared from *Clanis bilineata* larvae skin underwent demineralisation, deproteination, washing, drying, deacetylation and hydrolysis using commercial α-amylase, resulting in the acquisition of main products with a degree of polymerization in the range of 2–8 and an unexpected antibacterial activity [39]. A recombinant chitinase *LIChi18A* extracted from *Lactococcus lactis* and over-expressed in *Escherichia coli* BL21 (DE3) could also be used to produce COS from fungal waste mycelia [40]. Mallakuntla et al. extracted a single domain hyper-transglycosylating chitinase from *Enterobacter cloacae* subsp. *cloacae* 13047 (*EcChi1*). This chitinase could generate longer chitin oligosaccharides and the optimal conditions were 40 °C and pH 5.0 with k_cat_/K_m_ of 0.011 × 10^2^ (mg/mL)^−1^ min^−1^, and K_m_ of 15.2 mg·mL^−1^ [41]. Sánchez et al. prepared COS with two different approaches including one-step process (P1) and two-step process (P2). In P1, chitosan was dissolved in 0.2 M acetic/acetate buffer and depolymerized by chitosanases from *Streptomyces griseus*. In P2, chitosan was first dissolved in 0.1 M HCl and chemically hydrolyzed by KNO_2_. The solution was then precipitated by NH_4_OH and washed with continuous ethanol/distilled water. Finally, the resulting low-molecular-weight chitosan was depolymerized with the same chitosanase in P1. By structural analysis of products, the results indicated that the product COS was composed of 27% of fully deacetylated sequences obtained by P1 while 63% of fully deacetylated sequences by P2 [42]. Complex enzyme hydrolysis has more satisfactory efficiency than a single enzyme. Using complex enzymes composed of commercial α-amylase, cellulose and pectinase for chitosan hydrolysis, the products suggested a molecular weight below 4000 Da after 2 h, and more than 90% products had good water solubility without changing the chitosan glycosidic ring structure and deacetylation degree [43].

Enzymatic degradation has potential to produce chitosan oligomers with a fully defined structure, which is significant for mechanistically exploring their bioactivities. Hamer et al. adopted two chitin deacetylases including COD from *Vibrio cholera* and NodB from *Rhizobium* sp. GRH2 to produce defined chitosan oligomers in which the first two units beginning from the non-reducing end were deacetylated [44]. Hembach et al. reported that four tetramers out of 14 possible partially acetylated chitosan tetramers were purified (>95%) by fungal, bacterial and viral chitin deacetylases according to the degree of acetylation, degree of polymerization and pattern of acetylation [45]. These degradation methods effectively facilitate the research on relationships between structure and function.

### 2.4. Electrochemical Degradation

Electrochemical degradation is an advanced approach to prepare COS. This method is easy to operate and contamination-free, but there is still some problems such as the short electrode life and easy failure. It has been proved that the molecular weight of chitosan decreased with an increase in the current density of the Ti/TiO_2_-RuO_2_ electrode, while the protonated amino groups were stable during electrochemical treatment [46]. Furthermore, the Ti/Sb-SnO_2_ electrode was more effective than the Ti/TiO_2_-RuO_2_ electrode, due to the difference between the characteristics of electrode materials [47].

## 3. Analysis of COS

### 3.1. Chemical Component Analysis of COS

Chemical component analysis prominently facilitates the research on relationships between COS structure and function. Besides, these analytical techniques help us to figure out basic characteristics such as the degree of polymerization, the sequence of COS and so on [3]. Thin-layer chromatography (TIC) is an important experimental technique for rapid separation and qualitative analysis of substances with small amounts. Lee et al. used crude enzymes to hydrolyze chitosan, and then employed TIC with a mobile phase system composed of n-propanol–water–ammonia water (70:30:1, *v*/*v*/*v*) to separate products. Product analysis suggested that this method resulted in the production of glucosamine and COS with a degree of polymerization of 2–6 and above [48]. The same mobile phase system was also utilized by other researchers [49]. High performance liquid chromatography (HPLC) is also crucial for the degree of polymerization determination and quantitative analysis. Xu et al. prepared COS via enzymatic degradation, and the results from HPLC showed that the polymerization degree of products COS was 2–8 [50]. Mass spectrometry (MS), including electrospray ionization MS (ESI-MS), matrix-assisted laser desorption ionization time of flight MS (MALDI-TOF-MS), etc., is a common method for the analysis of oligosaccharides and has been widely used in the analysis of COS mixtures [51,52,53]. MS provides the molecular weight, polymerization degree, and sequences of COS and its derivatives [54,55]. In addition, Cordlandwehr et al. developed a promising method using MS which could be used for sequencing and quantitative analysis of partially acetylated COS (pa-COS). Firstly, a deuterated reagent was used to transform pa-COS into fully *N*-acetylated chitin oligomers; Secondly, complete quantification of pa-COS was analyzed by adding [^13^C, ^2^H]-labelled isotopologs as interior labels in LC-MS; Finally, quantitative measurement of pa-COS by tandem MS could be promoted by using the ^18^O-tag to label the reducing end [56].

### 3.2. Structural Analysis of COS

Structural analysis techniques including ultraviolet/visible (UV-Vis), infrared radiation (IR), nuclear magnetic resonance (NMR) spectroscopy and MS are widely used in COS structural research. IR spectroscopy could examine the physicochemical characterization and degree of *N*-acetylation of chitin or chitosan separately [57,58]. Since both *N*-Acetylglucosamine (GlcNAc) and GlcN residues have no interaction with the chitin/chitosan chain, the total absorbance of chitin/chitosan is only affected in a simple, additive way with residue addition. Thus, UV-Vis spectroscopy is able to determine the formation of chitin and chitosan by detecting GlcNAc and GlcN residues which are far-UV chromophoric groups [59]. In addition, various NMR spectroscopy could also be applied to study chitin, chitosan and its derivatives via ^13^C [60], ^1^H [61], ^31^P [62] NMR. However, NMR spectroscopy demands concentrated samples and COS with a degree of polymerization less than 5 [23]. Fortunately, there is an enzymatic/mass spectrometric fingerprinting approach whose accuracy is identical to NMR but requires a minute amount of sample [63]. Li et al. extracted crude chitosan from dry mycelia of *F. sambucinum* and obtained final mixtures successively by deacetylation, acid hydrolysis and purification. COS with a degree of polymerization of 5 (DP5) was analyzed via ESI-MS, Fourier transform infrared spectroscopy (FT-IR), and NMR. The results indicated that DP5 had a molecular weight of 823.33 and molecular formula of C_30_H_57_O_21_N_5_. Moreover, types of chemical bonds and distribution of C-H bond in DP5 were also determined [64]. Methods for determining the physicochemical properties of chitin, chitosan, and COS are summarized in Table 2.

## 4. Biological Functions of COS

### 4.1. Antioxidant Activity

Oxidative stress is affiliated with multifarious degenerative disease such as cancer, macular degeneration, and arteriosclerosis [3]. Studies have shown that COS and its derivatives have a high total reducing power and can effectively scavenge hydroxyl radicals and superoxide anions [65,66]. COS is able to stop the free radical chain reaction since COS can provide positrons to free radicals and convert free radicals into more stable products. Meanwhile, the ability of COS to form intramolecular hydrogen bonds remains as the involved functional groups (i.e., hydroxyl and amino groups) are still present in the oligosaccharide, and the resulting force of union between molecules is reduced proportionally to the chain length. Thus, many active hydroxyl groups and amino groups are easily activated after they are exposed, which helps to scavenge free radicals [67]. Novel COS derivatives are synthesized by linking COS and gallic acid reduced oxidative stress by increasing the expression levels of antioxidant enzymes like glutathione (GSH) and superoxide dismutase (SOD) [68]. Huang et al. used SH-SY5Y cells to investigate the effect of COS on Cu^2+^-induced oxidative damage and suggested that COS could downregulate the levels of cellular oxidative stress [69]. Besides, 4-hydroxybenzaldehyde-COS (HB-COS) was synthesized and proved to significantly decrease reactive oxygen species generation and upregulate the protein levels of antioxidative enzymes such as heme oxygenase-1 (HO-1) and catalase (CAT) [70]. COS also obviously alleviated H_2_O_2_-induced oxidative stress in ECV304 cells [71]. Xie et al. [72] employed pregnant sows to explore the supplement effect of COS on antioxidant defense. The results indicated that with COS supplementation, plasma total SOD as well as the mRNA expression of antioxidant genes in the placenta was increased, while plasma malondialdehyde (MDA) was reduced.

Moreover, COS could enhance defense response of plants by modulating the action of antioxidant enzymes. Zou et al. used wheat seedlings to explore the potential protective effect of sulfated chitooligosaccharide (SCOS) on the defense response of plants under salt stress. Their results indicated that by regulating the activities of antioxidant enzymes, SCOS could relieve the damage of salt stress in wheat seedlings possibly due to its sulfate group [73]. Cadmium (Cd) is a heavy metal, which has detrimental effects on crop quality and yields. Edible rape was exposed to a Hoagland nutrient solution which contained 50 μM Cd, and then COS was sprayed onto the leaves of edible rape. Compared with the control group, the edible rape exposed to COS presented a lower concentration of Cd^2+^ in shoots and roots. Meanwhile, the activities of SOD and CAT in COS treated group were significantly enhanced, suggesting the potential of COS in improving plant resistance to Cd was possibly due to promoting antioxidant enzyme activities [74]. Vander et al. evaluated the capability of GlcNAc (tetramer to decamer) and of GlcN (pentamer and heptamer) and partially *N*-acetylated chitosans to induce phenylalanine ammonia-lyase (PAL), peroxidase (POD) as well as lignin deposition in healthy wheat leaves, and their results indicated that different mechanisms are involved in the induction of POD activities by GlcNAc oligomers, and PAL and POD activities by partially *N*-acetylated chitosan polymers [75].

### 4.2. Antitumor Activity

Studies on the effectiveness of COS in reducing cancer cells growth were reported as early as 1970s. The mechanism of anticancer activity was first considered to be attributed to the cationic nature of COS, and later relative molecular weight was also speculated to be one of important reasons [76,77,78]. Ronghua et al. verified the relationship between charge properties and anticancer activity of COS among three cell lines, HeLa, Hep3B and SW480. The result suggested that highly charged COS could significantly lower cancer cells viability regardless of positive or negative charge of COS [79]. A549 cells were also used to evaluate the antitumor activity of five chitooligomers that ranging from the dimer to the hexamer, and the result suggested that chitohexaose exhibited the most repressive effect on A549 cells proliferation. Further, chitohexaose could also downregulate expression levels of *cyclin D1* and *bcl-xl* which are closely related to cell cycle as well as apoptosis [80].

THP-1 cell line was also employed to evaluate anticancer activities of chitin, chitosan and low molecular weight chitin. The results indicated that when the concentration equaled or more than 1500 μg/mL, chitin and chitosan were able to inhibit 100% of the growth of THP-1 tumor cells, while low-molecular-weight chitin at the concentration of 250 μg/mL showed the same effect, suggesting the negative correlation between tumor inhibitory effect and the molecular weight of chitin [81]. Modified COS named aminoethyl-chitooligosaccharides (AE-COS) was synthesized by substituting hydroxyl groups with aminoethyl group at C-6 position. The results from MTT assay, gelatin zymography and western blot investigating the expression levels of MMP-2 and MMP-9, which are related to invasion of cancer cells, suggested that AE-COS could prevent cells from invasion associated with metastasis [82].

### 4.3. Antimicrobial Activity

COS is a good natural antimicrobial agent that can inhibit the growth of a variety of food pathogenic microorganisms, plant pathogenic bacteria, animal pathogens bacteria, and viruses. In the 1980s, chitosan and its derivatives were first reported to have extensive antimicrobial properties [83]. The antimicrobial activity of COS is related to the molecular weight, pH, degree of deacetylation, chemical modification, etc. Jeon et al. examined the antimicrobial effect of COS with relatively higher, medium and lower molecular weight, respectively, and the results indicated that COS with higher molecular weight showed better inhibitory effect than others [84]. However, these results may be contradicted according to the source and preparation methods of COS, target microorganism and experimental conditions, since these factors could result in different by-products as well as differentiation in the chemical structure of terminal groups of polysaccharide chains [85]. Lower pH value and higher polymerization were also reported to be beneficial due to the antimicrobial activity of COS [86]. Rúnarsson et al. proved that *N*-quaternization of COS contributes to antimicrobial activity under neutral conditions, and the efficiency of its antimicrobial activity could be enhanced with increasing alkyl chain length of the quaternary amine [87,88]. With addition of enteropathogenic *Escherichia coli* (EPEC) to the surface of the human HEp-2 cell line, Quinterovillegas et al. reported that COS could suppress the adherence of EPEC to HEp-2 by more than 90% [89]. Moreover, COS has great potential to develop novel antifungal drugs. The dermatophytic fungus *Trichophyton rubrum* were used to verify the antifungal activity of COS, and the results showed that *T. rubrum* cell growth was significantly suppressed when cells were treated with 0.5% and 1% COS. Besides, no inflammatory reaction or tissue injury was observed when the concentration of COS was as high as 5% [90]. The development of *Ustilago maydis* could also be inhibited by COS [91]. COS was reported to prolong the shelf-life of beer because of its good inhibitory effect on beer-spoilage bacteria where COS with the molecular weight of around 2 kDa exhibited optimal effects [92]. COS could also prolong the shelf-life of minced pork because it can inhibit the activities of Gram-positive and Gram-negative microbes effectively [93].

### 4.4. Immunomodulatory and Anti-Inflammatory Function

The immunomodulatory effect of COS is related to immune organs or cells that induce the secretion of cytokines. Wei et al. found that a high concentration of COS could promote the proliferation of bone marrow cells and induce CD34+ cells into megakaryocytes progenitor cells via facilitating stromal cell secretion of hematopoietic growth factors. Meanwhile, the bone marrow could differentiate into a variety of immune cells and enhance the immunity level of the body [94]. Macrophages isolated from blunt snout bream were used to verify the immunomodulatory property of COS, and the result implied that COS could improve the phagocytic activity of macrophages possibly due to increased gene expression of nicotinamide adenine dinucleotide phosphate (NADPH) oxidase, inducible nitric oxide synthase (iNOS), interleukin (IL)-1β and tumor necrosis factor –*α* (TNF-*α*) [6].

The immunomodulatory effect of COS is also related to its deacetylation degrees. Different deacetylation degrees of COS were used in the conjugation to porcine circovirus type 2 (PCV2) vaccines by covalent linkage, and the vaccine conjugates were administered to BALB/c mice to enhance immunogenicity. The results indicated that COS-PCV2 conjugates significantly enhanced both humoral and cellular immunity by increasing lymphocyte proliferation and initiating a mixed T-helper 1 (Th1)/T-helper 2 (Th2) response. Moreover, both immune responses to PCV2 and the adjuvant effect were positively related to the deacetylation degree of COS [95]. Dietary supplementation of COS was proved to show strong immunoenhancing activity in animals. Mei et al. evaluated the effect of COS on cyclophosphamide (Cy)-induced immunosuppression, and the results implied that delayed-type hypersensitivity (DTH) reaction, macrophage phagocytosis activities, and the levels of cytokines IL-2, IL-12 as well as interferon (IFN)-γ were significantly enhanced while the production of IL-10 was reduced in mice orally treated with COS plus Cy than those exposed to Cy alone [96]. Nguyen et al. reported that a remarkable decrease in mortality and enhanced phagocytic activity were observed in striped catfish fed with 100–200 mg COS per kg for 45 days [97]. In continuous feeding of *Paralichthys olivaceus* with supplementation of 0.5% or 1% COS diets for 28 days, Li et al. proved that the leukocyte phagocytic rate, phagocytic index, serum lysozyme activity and the number of peripheral leukocytes were significantly increased. In addition, COS enhanced the non-specific immune response and improved survival rates upon challenge with *Edwardsiella tarda* in *P. olivaceus* [98]. Yin et al. [99] found that dietary replenishment of COS resulted in enhancing serum levels of IL-1β, IL-2, IL-6, immunoglobulin (Ig) A, IgG and IgM in early-weaned piglets. Their findings suggested that COS could improve cell-mediated immune response under wean stress by regulating the levels of particular cytokines and antibodies.

Inflammation plays a significant role in the pathology of numerous diseases such as cancer, autoimmune diseases and cardiovascular disease [100,101,102]. It is proved that activation of myeloperoxidase, cyclooxygenase (COX)-2 and iNOS as well as the levels of pro-inflammatory cytokines such as IL-6 and TNF-α were suppressed by oral administration of COS [103]. In addition, COS could exert anti-inflammatory effect by remarkably decreasing the levels of NO, TNF-α and IL-1β resulting in the repression of NF-κB pathway [104]. Glycoprotein YKL-40 (also called HC-gp39), a biomarker of inflammation, is an inactive chitinase of chitin proteinase family 18. Partially acetylated *N*-acetylglucosamine could bind to YKL-40 with high-affinity and stimulate chondrocyte proliferation, suggesting an innovative approach to cure inflammatory rheumatoid diseases [105]. Besides, YKL-40 was reported to act as a negative regulator of the inflammasome and promote the occurrence of angiogenesis. Gudmundsdottir et al. concluded that the proinflammatory mechanism of chitosan or highly deacetylated chitosan oligosaccharide was partly due to reducing the secretion of YKL-40 [106].

### 4.5. Neuroprotective Effect

**Figure 2:**
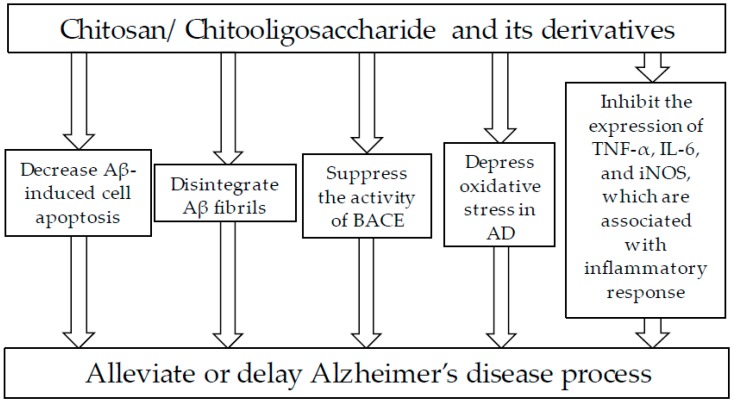
The neuroprotective effect of COS against Alzheimer’s disease.

The prevalence of neurodegenerative disorders including Alzheimer’s disease (AD) is the most urgent problem for seniors in the world [107]. Neuropathological hallmarks of AD are the presence of intracellular neurofibrillary tangles and extracellular amyloid plaques [108]. The classic hypotheses of AD include β-amyloid (Aβ) accumulation, acetylcholine (ACh) deficiency, increased inflammatory response and so on [109]. Dai et al. have made plenty of efforts to explore the neuroprotective effect of COS against AD. They reported that COS significantly decreased Aβ-induced cell apoptosis by reducing the expression of caspase-3 and Bax/Bcl-2 ratio activation [110]. Besides, their results suggested that COS could disintegrate Aβ_1-42_ fibrils formation, implying that COS has both anti-Aβ fibrillogenesis and fibril-destabilizing ability [111]. Recently, Dai et al. proposed that COS exerted its neuroprotective effect partly through suppressing β-site amyloid precursor protein-cleaving enzyme 1 (BACE1) expression and enzymatic activity [112]. Eom et al. prepared various phenolic acid-conjugated COS like hydroxyl cinnamic acid and hydroxyl benzoic acid to explore their inhibitory effect on BACE, and the results indicated that caffeic acid-conjugated COS was most effective in repressing BACE and ameliorating AD [113]. Jia et al. established an Aβ_1-42_ rat model of AD and orally administrated rats with COS of various concentrations for 15 days in order to verify the effect of COS on AD. The results suggested that COS could ameliorate the cognitive impairments by suppressing oxidative stress and neuroinflammatory responses [114]. Kim et al. used the human astrocytoma cell line to explore the effect of water-soluble chitosan (WSC) on inflammatory response associated with Aβ and IL-1β in AD. The results showed that WSC was able to inhibit the expression of TNF-α, IL-6, and iNOS in human astrocytoma cells stimulated by IL-1β or Aβ peptide [115]. The possible neuroprotective effect of COS against AD is summarized in Figure 2.

In a mouse model of sciatic nerve injury, COS could suppress scar formation and improve function recovery by altering the proportion of various vascular diameters and inducing fibroblast death [116]. Yoon et al. employed three derivatives of COS including aminoethyl (AE)-COS, dimethylaminoethyl (DMEM)-COS and diethylaminoethyl (DEAE)-COS to explore their effects on cholinesterase, and the result proved that DEAE-COS was most efficient in depressing AChE activity [117]. In addition, there is increasing evidence suggesting that COS could exert neuroprotective effects by inhibiting oxidative stress and neuroinflammation [118,119]. Zhao et al. proved that COS could stimulate nerve regeneration since the axis of miR-327/CCL2 in Schwann cells was identified as a potential target of COS according to transcriptome analysis. They confirmed that COS could stimulate CCL2 expression by down-regulating miR-327 in Schwann cells which induces macrophage migration at injury sites to reconstruct microenvironments and thus promote nerve regeneration [120].

### 4.6. Medical Application as Auxiliary Materials

Since COS has good water-solubility, it is sometimes employed as supplementary materials to promote drug efficacy. Curcumin has special anticancer activities though systemic bioavailability has been limited due to its instability and insolubility in water. An innovative formulation encapsulating curcumin in stearic acid-g-chitosan oligosaccharide (CSO-SA) polymeric micelles was designed to promote curcumin accumulation in cancer cells and suppress subpopulations of CD44+/CD24+ cells, which implied that COS could enhance the anticancer properties of curcumin [121]. Stricker-Krongrad et al. [122] investigated the hemostatic efficacy of the chitosan related Opticell dressing in heparinized rats which had excisional wounds that imitating debridement. Results from total bleeding and rate of bleeding in rats suggested that topical application of Opticell dressing with chitosan could be an available method in controlling bleeding correlated with wound debridement. COS was also used to design membrane films by blending various concentrations of glycerol with COS, where the membrane film exhibited high water vapor transmission rates and water absorption capacity thus could be applied to wound-dressing materials [123]. Decanoic acid grafted oligochitosan nanoparticles (CSO-DA NPs) was designed to explore its potential as a carrier for insulin. The results showed that hypotoxic 50 IU/kg dose of CSO-DA NPs decreased the serum glucose level by 57.18% [124]. In conclusion, COS has a promising prospect in medical application as auxiliary materials.

### 4.7. Other Bioactivities

In addition to the effects mentioned above, COS has various novel functions as described below. In common organic solvent, *tert*-Butyldimethylsilyl O-protected COS and chitosan are available precursors for *N*-modifications [125]. Carboxymethyl-quaternary ammonium oligochitosan (CM-QAOC) could be used as a fluorescent tracing chemical for industrial cooling water treatment since the fluorescence of CM-QAOC was not affected by common phosphorus-containing inorganic and organic water treatment chemicals or *N*-dodecyl-*N*,*N*-dimethyl-benzenemethanaminium chloride but was influenced by metal ions such as Fe^3+^ and Cu^2+^ from raw water or corrosion products [126]. Zhang et al. evaluated hypolipidemic activities of high- (712.6 kDa) or low- (39.8 kDa) molecular-weight chitosan in high-fat diet fed rats, and the results indicated that hypolipidemic activities of low-molecular-weight chitosan was better than high-molecular-weight chitosan, possibly due to higher serum and liver lipoprotein lipase activities [127]. Chiu et al. also reported that low-molecular-weight chitosan exerted a significant effect in improving the activity of lipid metabolism and intestinal disaccharidase in obese rats with a high-fat diet [128]. Besides, COS was also reported to be resistant to tobacco mosaic virus (TMV) in *Arabidopsis* via stimulation of the salicylic acid signaling pathway [129]. Chen et al. [130] reported that fully deacetylated COS (GlcN)_2__–7_ was able to inhibit the activities of the Glycoside hydrolase family 18 chitinase, including the human chitinase *HsCht* and the insect chitinase *OfChtI*, with IC_50_ values at micromolar to millimolar levels. COS was also reported to have potential in helping wound healing. Li et al. prepared a silver nanoparticle/COS/ poly (vinyl alcohol) (PVA/COS-AgNPs) nanofiber via electrospinning and explored the mechanism underlying the accelerated healing effect of the PVA/COS-AgNPs nanofiber in wounded rats. The results suggested that the mechanism was possibly related to upregulated expression levels of cytokines associated with the TGFβ1/Smad signal pathway, including TGFβ1, TGFβR1, collagen I, pSmad 2, etc. [131]. COS could regulate the gene expression of hMSC and the secretion of cytokine like IL-6 and IL-8 during osteogenic differentiation but not mineralization. Meanwhile, *N*-Acetyl chitohexaose had stronger effects on cell stimulation than chitohexaose, suggesting the effect of COS was partly dependent on the acetylation degree [132]. Besides, supplying ICR mice with a 30 mg/kg dose of chitosan supplements for two weeks did not affect growth performance but impaired intestinal barrier integrity manifested by increased serum D-lactate content and decreased jejunal diamine oxidase activity. Moreover, the gene expression of intestinal tight junction proteins that are indispensable to maintain tight junction stability and barrier function including occludin and ZO-1 was significantly reduced due to compromised intestinal barrier integrity [133].

## 5. Conclusions

The preparation, analysis and biological functions of COS have been summarized in this review. High solubility, low molecular weight, strong absorption capacity and good biocompatibility render COS various bioactivities such as antitumor and neuroprotective effects, immunomodulatory functions as well as antioxidant activities. However, there are still several problems that should be resolved. COS was reported for its wide use in pharmacy and biomedicine, but no long-term safety studies on humans have been carried out to our knowledge. Besides, both molecular weight and deacetylation degrees affect the performance of COS in drug delivery. However, COS produced by present methods has different molecular weights, degrees of polymerization, structures and physico-chemical characteristics, which may result in great changes in biological constituents and bioactivities. Therefore, it is important to ensure reproducible processes that produce well-defined COS and establish methods for physicochemical characterization to extend the understanding of its bioactivity. Besides, single component COS is also significant in studying the mechanism and broadening the range of applications though it demands higher standards for preparation methods. Further research can optimize the preparation process and develop better methods to generate single component COS. Moreover, further research and development on innovative COS derivatives like carboxylated, sulfated as well as phenolic acid conjugated COS and their applications in functional foods, nutraceuticals, and pharmacy could substantially enhance the stability and bioavailability of COS.

## Figures and Tables

**Table 1:** The advantages and disadvantages of various preparation methods of COS.

Preparation Methods of COS	Advantages	Disadvantages
Chemical degradation	Simple to handle	Difficult to separate and purify the products
Physical degradation	Easy purification and little contamination	Low productivity
Enzymatic degradation	Easy accessibility, no additional product modifications	High cost but low availability
Electrochemical degradation	Easy to operate and contamination-free	Short electrode life and easy to fail

**Table 2:** Methods for determining the physicochemical properties of chitin, chitosan, and COS.

Physicochemical Properties	Determination Methods
Degree of deacetylation	Potentionmetric method [17,33,34] FT-IR [25] Acid-base titration [26]
Degree of polymerization	MALDI-TOF MS [18], ^1^H NMR [20]
Degree of *N*-acetylation	MALDI-TOF MS and ^1^H NMR [18] HPLC-a photodiode array (PDA) [23]
Chemical structure	FT-IR [22,30,33,34] ESI-MS [31] UV [33,34]
Viscosity	A falling-ball viscometer [24]
Color tone intensity	Etereo microscope [25]
Intrinsic viscosity molecular weight	An Ubbelohde Viscometer [25,26,32,34]

## Abbreviations

ACh	Acetylcholine
AD	Alzheimer’s disease
AE-COS	Aminoethyl-chitooligosaccharides
Aβ	β-Amyloid
BACE	β-Site amyloid precursor protein-cleaving enzyme
CAT	Catalase
Cd	Cadmium
CM-QAOC	Carboxymethyl-quaternary ammonium oligochitosan
CNP	Chitosan nanoparticles
COS	Chitooligosaccharide
COX	Cyclooxygenase
CSO-DA NPs	Decanoic acid grafted oligochitosan nanoparticles
CSO-SA	Stearic acid-g-chitosan oligosaccharide
Cy	Cyclophosphamide
DEAE	Diethylaminoethyl
DE3	*Escherichia coli* BL21
DMEM	Dimethylaminoethyl
DTH	Delayed-type hypersensitivity
EEP	Extracellular enzymes preparation
EPEC	Enteropathogenic *Escherichia coli*
ESI	Electrospray ionization
GlcN	Glucosamine
GlcNAc	*N*-Acetylglucosamine
GSH	Glutathione
HB-COS	4-Hydroxybenzaldehyde-COS
HO-1	Heme oxygenase-1
HPLC	High performance liquid chromatography
IFN	Interferon
Ig	Immunoglobulin
iNOS	Inducible nitric oxide synthase
IL	Interleukin
IR	Infrared
MALDI-TOF	Matrix-assisted laser desorption ionization time of flight
MDA	Malondialdehyde
MS	Mass spectrometry
NADPH	Nicotinamide adenine dinucleotide phosphate
NMR	Nuclear magnetic resonance
Pa-COS	Partially acetylated COS
PCV2	Porcine circovirus type 2
PVA/COS-AgN	Silver nanoparticle/COS/ poly (vinyl alcohol)
SCOS	Sulfated chitooligosaccharide
SOD	Superoxide dismutase
TIC	Thin-layer chromatography
UV-Vis	Ultraviolet/visible
WSC	Water-soluble chitosan
